# Supplementary material for: The Systems Biology Research Tool: evolvable open-source software
Source: BMC Syst Biol. 2008 Jun 29;2:55. doi: 10.1186/1752-0509-2-55 (PMC2446383; doi:10.1186/1752-0509-2-55)
Supplement: Additional file 1 — SBRT Archive. An archive of the current version of the Systems Biology Research Tool. [file 1752-0509-2-55-S1.zip › sbrt-1.4.0/doc/developers_guide/api/sbrt/ext/R/DummyConsole.html]

DummyConsole


|  |  |  |
| --- | --- | --- |
| **Overview** \| **Package** \| **Class** \| **Use** \| **Tree** \| **Deprecated** \| **Index** \| **Help** | |  |
| PREV CLASS   **NEXT CLASS** | **FRAMES**    **NO FRAMES**     **All Classes** |  |
| SUMMARY: NESTED \| FIELD \| CONSTR \| METHOD | DETAIL: FIELD \| CONSTR \| METHOD |  |


---


## sbrt.ext.R Class DummyConsole

```
java.lang.Object
  sbrt.ext.R.DummyConsole
```

**All Implemented Interfaces:**: org.rosuda.JRI.RMainLoopCallbacks

---

``` public class DummyConsole extends java.lang.Object implements org.rosuda.JRI.RMainLoopCallbacks ```

This class is used to make dummy consoles for the JRI.

**Author:**
:   This class was written and documented by
    Jeremiah Wright while in the Wagner lab.

---

**Constructor Summary**

`DummyConsole()`


| **Method Summary** | |
| --- | --- |
| `void` | `rBusy(org.rosuda.JRI.Rengine re, int which)`             Does nothing. |
| `java.lang.String` | `rChooseFile(org.rosuda.JRI.Rengine re, int newFile)`             Does nothing. |
| `void` | `rFlushConsole(org.rosuda.JRI.Rengine re)`             Does nothing. |
| `void` | `rLoadHistory(org.rosuda.JRI.Rengine re, java.lang.String filename)`             Does nothing. |
| `java.lang.String` | `rReadConsole(org.rosuda.JRI.Rengine re, java.lang.String prompt, int addToHistory)`             Does nothing. |
| `void` | `rSaveHistory(org.rosuda.JRI.Rengine re, java.lang.String filename)`             Does nothing. |
| `void` | `rShowMessage(org.rosuda.JRI.Rengine re, java.lang.String message)`             Does nothing. |
| `void` | `rWriteConsole(org.rosuda.JRI.Rengine re, java.lang.String text, int oType)`             Does nothing. |

**Methods inherited from class java.lang.Object**

`clone, equals, finalize, getClass, hashCode, notify, notifyAll, toString, wait, wait, wait`

**Constructor Detail**

### DummyConsole

```
public DummyConsole()
```


**Method Detail**

### rWriteConsole

```
public void rWriteConsole(org.rosuda.JRI.Rengine re,
                          java.lang.String text,
                          int oType)
```

:   Does nothing.

    :   **Specified by:**: `rWriteConsole` in interface `org.rosuda.JRI.RMainLoopCallbacks`
    :   **Parameters:**: `re` -: `text` -: `oType` -

---


### rBusy

```
public void rBusy(org.rosuda.JRI.Rengine re,
                  int which)
```

:   Does nothing.

    :   **Specified by:**: `rBusy` in interface `org.rosuda.JRI.RMainLoopCallbacks`
    :   **Parameters:**: `re` -: `which` -

---


### rReadConsole

```
public java.lang.String rReadConsole(org.rosuda.JRI.Rengine re,
                                     java.lang.String prompt,
                                     int addToHistory)
```

:   Does nothing.

    :   **Specified by:**: `rReadConsole` in interface `org.rosuda.JRI.RMainLoopCallbacks`
    :   **Parameters:**: `re` -: `prompt` -: `addToHistory` - **Returns:**: `null`.

---


### rShowMessage

```
public void rShowMessage(org.rosuda.JRI.Rengine re,
                         java.lang.String message)
```

:   Does nothing.

    :   **Specified by:**: `rShowMessage` in interface `org.rosuda.JRI.RMainLoopCallbacks`
    :   **Parameters:**: `re` -: `message` -

---


### rChooseFile

```
public java.lang.String rChooseFile(org.rosuda.JRI.Rengine re,
                                    int newFile)
```

:   Does nothing.

    :   **Specified by:**: `rChooseFile` in interface `org.rosuda.JRI.RMainLoopCallbacks`
    :   **Parameters:**: `re` -: `newFile` - **Returns:**: `null`.

---


### rFlushConsole

```
public void rFlushConsole(org.rosuda.JRI.Rengine re)
```

:   Does nothing.

    :   **Specified by:**: `rFlushConsole` in interface `org.rosuda.JRI.RMainLoopCallbacks`
    :   **Parameters:**: `re` -

---


### rLoadHistory

```
public void rLoadHistory(org.rosuda.JRI.Rengine re,
                         java.lang.String filename)
```

:   Does nothing.

    :   **Specified by:**: `rLoadHistory` in interface `org.rosuda.JRI.RMainLoopCallbacks`
    :   **Parameters:**: `re` -: `filename` -

---


### rSaveHistory

```
public void rSaveHistory(org.rosuda.JRI.Rengine re,
                         java.lang.String filename)
```

:   Does nothing.

    :   **Specified by:**: `rSaveHistory` in interface `org.rosuda.JRI.RMainLoopCallbacks`
    :   **Parameters:**: `re` -: `filename` -


---


|  |  |  |
| --- | --- | --- |
| **Overview** \| **Package** \| **Class** \| **Use** \| **Tree** \| **Deprecated** \| **Index** \| **Help** | |  |
| PREV CLASS   **NEXT CLASS** | **FRAMES**    **NO FRAMES**     **All Classes** |  |
| SUMMARY: NESTED \| FIELD \| CONSTR \| METHOD | DETAIL: FIELD \| CONSTR \| METHOD |  |


---
